# *Hepatocystis* and *Nycteria* (Haemosporida) parasite infections of bats in the Central Region of Cameroon

**DOI:** 10.1017/S0031182021001542

**Published:** 2022-01

**Authors:** K. J. A. Tsague, E. M. Bakwo Fils, J. P. Atagana, N. V. Dongue, D. W. Mbeng, J. Schaer, T. Tchuinkam

**Affiliations:** 1 Laboratory of Biological Sciences, Faculty of Sciences of University of Maroua, Maroua, Cameroon; 2 Vector Borne Diseases Laboratory of the Research Unit for Biology and Applied Ecology (VBID-RUBAE), University of Dschang, Dschang, Cameroon; 3 Department of Molecular Parasitology, Institute of Biology, Humboldt University, Berlin, Germany

**Keywords:** Bats, Chiroptera, Haemosporidia, *Hepatocystis*, *Nycteria*

## Abstract

Mammalian haemosporidian parasites are classified in ten genera, including *Plasmodium*, *Hepatocystis* and *Nycteria*. A high diversity of haemosporidian parasites has been described from bats, but our understanding of their prevalence, distribution and use of hosts remain fragmented. The haemosporidian parasites of bats in Cameroon have been largely understudied, but here, bats, sampled from different habitat types of the Central Region of Cameroon, were investigated for haemosporidian infections with a combination of microscopic and molecular phylogenetic analysis. An overall prevalence of 18.1% of haemosporidian infections was detected in a total of 155 investigated bats belonging to 14 bat species. For the first time *Hepatocystis* and *Nycteria* parasites were detected in bats from Cameroon and molecularly characterized. *Hepatocystis* infections were exclusively identified in the epauletted fruit bat host species *Epomophorus pusillus* with a high prevalence of 65.5%, whereas *Nycteria* infections could be detected in several hosts, namely: *Doryrhina cyclops* (60.0%), *Rhinolophus landeri* (20.0%) and one *Nycteris grandis*. This study unveils evidence that habitat types may play a role in transmission of *Hepatocystis* parasites on a local scale and it adds important information on the distribution and host specificity of the neglected haemosporidian genus *Nycteria.*

## Graphical abstract

**Figure:**
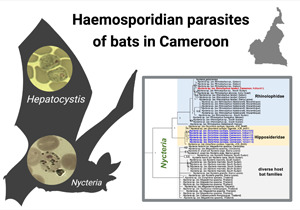

## Introduction

Haemosporidian parasites (phylum Apicomplexa) infect a wide range of mammals including primates, rodents and bats (Garnham, 1966). The human-infecting *Plasmodium* species belong to a large group of haemosporidian parasites of about 500 closely related species (Martinsen and Perkins, 2013; Galen *et al*., 2018). These parasites use a diverse array of dipteran and vertebrate hosts to complete their life cycle, the latter comprising birds, saurian reptiles and mammals (Garnham, 1966; Levine, 1988). Mammalian haemosporidian parasites are classified in ten different genera, including *Plasmodium*, *Hepatocystis* and *Nycteria* (Perkins and Schaer, 2016). Previous studies led to the discovery of several parasite lineages in bats and revealed unexpected phylogenetic relationships (e.g. Duval *et al*., 2007; Schaer *et al*., 2013), which suggests that bats have played an important role in the evolutionary history of malaria parasites (Perkins and Schaer, 2016; Galen *et al*., 2018). A broader sampling and systematic analysis of bat malaria parasites is essential for better understanding the evolutionary history of haemosporidian parasites, including the human-infecting species (Perkins and Schaer, 2016).

Parasites of the genus *Hepatocystis* infect a wide range of vertebrate hosts including primates, bats, ungulates and rodents, whereas *Nycteria* parasites exclusively infect insectivorous bats (Garnham, 1966). Historical classification of mammalian haemosporidian parasites was done according to morphological and biological characteristics, resulting in initial misplacement of some chiropteran haemosporidian parasites within the genus *Plasmodium*. However, the observation that certain bat parasites lack schizogony in erythrocytes, and therefore cannot represent the genus *Plasmodium*, led to a reclassification of several species, which was later confirmed by molecular phylogenetic studies (e.g. Garnham, 1950; Perkins, 2014). The mammalian *Plasmodium* clade is paraphyletic as it contains the parasites of the genus *Hepatocystis* (Galen *et al*., 2018). The dipteran vectors for most chiropteran haemosporidian parasites, including *Nycteria,* remain unknown (Schaer *et al*., 2013, 2015).

Bats are the only flying mammals and exhibit a very high species diversity (Arthur and Lemaire, 1999; Simmons and Cirranello, 2020). They have unique characteristics among mammals and have an ecological diversity of niches that makes them key organisms in maintaining the ecosystem balance (Reis and Guillaumet, 1983; Rodriguez *et al*., 2006; Aziz *et al*., 2021). Indeed, numerous studies have demonstrated the important role of the Chiroptera in insect population regulation, pollination and seed dispersal of many ecologically and economically important plants (Tchatat, 1999; Hutcheon, 2003; Bakwo Fils, 2009; Kunz *et al*., 2011; Bakwo Fils *et al*., 2012; Ingala *et al*., 2021). This is especially the case in tropical areas, where these animals seem to be among the main agents of seed dissemination due to their abundances and their activities (Reis and Guillaumet, 1983).

This study presents a survey of haemosporidian parasite infections in different bat species in the central region of Cameroon and determines the prevalence, parasitaemia and phylogenetic relationships of haemosporidian infections in bats.

## Materials and methods

**Fig. 1.**
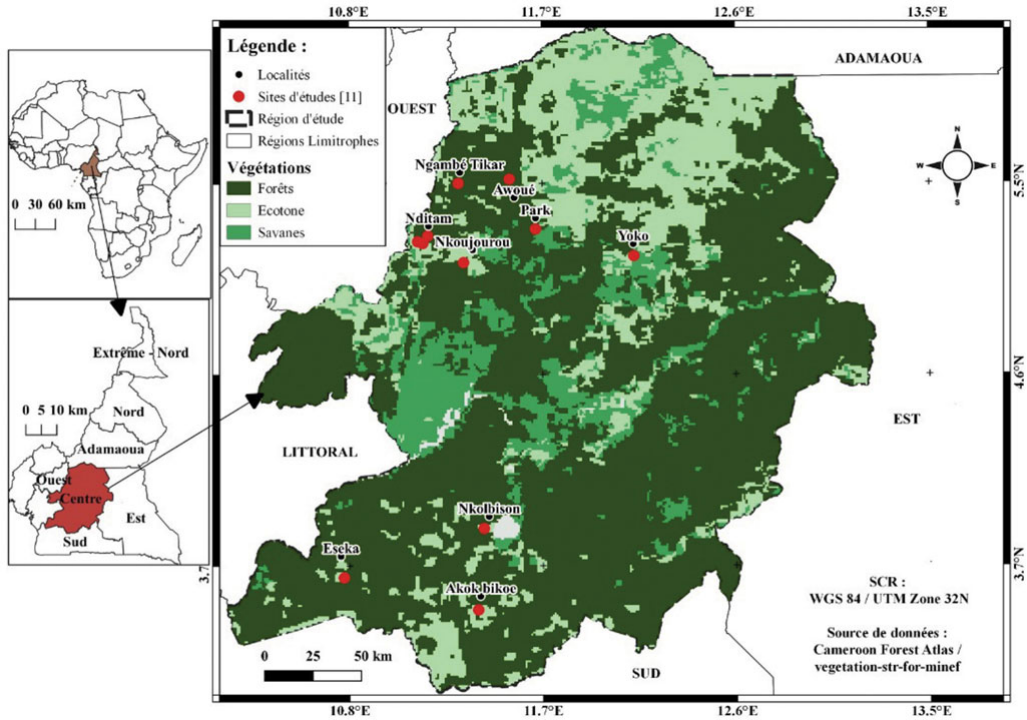
(A) Map of central region of Cameroon depicting the sampling sites (sampling sites are marked with red dots).

Bats were sampled in the central region of Cameroon between February 2016 and December 2019 representing both dry and wet seasons. Sampling sites covered the different habitat types: forest, savanna and cultured farmland (Fig. 1). The prevailing climate is equatorial of Guinea type with dry seasons in July to August and December to March and rainy seasons from April to June and September to November (Maurice *et al*., 2011). Temperatures are moderate to high, and constant (mean temperature = 28°C). Precipitation varies between 1500 mm and 3000 mm per year. Vegetation in the region is dominated by a dense and humid tropical forest. Soils are clay and red. The river Nyong (750 km long) is the main water stream of the region.

### Field sampling and microscopy

Bats were captured using ground-level mist nets. Mist nets were set every capture night from 6 pm to 5 am across potential flight trajectories of bats: above water points, in clearings of farmland, in widespread savannah, in the forest. Sampling was also carried out in diurnal roosts like caves, tunnels, abandoned houses. Standard measurements like sex, age, forearm lengths were recorded, and species were identified using different identification keys (Rosevear, 1965; Hayman and Hill, 1971; Patterson and Webala, 2012; Happold and Happold, 2013). The bats were released, but one adult female and one adult male per species were chosen as voucher specimens and preserved in 70% (vol/vol) ethanol and accessioned in the Biological Laboratory Museum of the University of Maroua.

Blood samples of 10–30 *μ*L were collected from each individual by venipuncture of the uropatagial vein. After the blood collection, cotton was placed on the haemostatic vein until the bleeding had stopped; then the individual was released. The blood sample was used to prepare a thin and a thick blood smear shortly after collection. The blood smears were dried and fixed in 99–100% (vol/vol) methanol solution for 3 s. All blood smears were stained with 10% Giemsa solution for 15 to 20 min and dried. The remaining blood sample was dotted on Whatman filter papers. Blood smears and blood dots were immediately air-dried in humid environment in the field with a battery-operated fan and then the samples were dried for at least 3 h in an environment with low humidity. After drying, the filter cards were stored in Ziploc bags containing a sachet with desiccant (silicate gel) and subsequently stored in a freezer at −20°C.

Giemsa-stained blood smears were examined for the presence and parasitemia of haemosporidian parasites and for the analysis of the morphology of observed parasite stages for 20–40 min, using a light microscope (Leica DMLB 1000) at a magnification of ×1000 with immersion oil.

Parasitaemia values (% of infected erythrocytes) were calculated for all bats with confirmed haemosporidian infections. The mean number of erythrocytes per field was determined by counting them in 1–3 fields, and the number of parasites was recorded in 20–100 fields. For this purpose, fields with comparable erythrocyte density were chosen (Schaer *et al*., 2015). Parasitaemia (in percent) was determined following the calculation: total number of parasites/mean number of erythrocytes per field × number of counted fields.

### Molecular methods

DNA was extracted from the dried blood dots on Whatman filter paper (GE Healthcare) using the QIAGEN DNeasy extraction kit (Hilden, Germany) (e.g. Schaer *et al*., 2015). The protocol for animal tissues was performed with the minor modification of elution of the samples in 50–100 *μ*L AE buffer depending on the density of the blood dot. PCRs were performed using the AllTaq Master Mix Kit (QIAGEN) with 4–5 *μ*L of genomic DNA as the template, and 1 *μ*L of each primer (10 mm). Five genes from the three parasite genomes were targeted for detection and subsequent phylogenetic analysis of haemosporidian parasites: the mitochondrial genes cytochrome b (*cytb*) and cytochrome oxidase 1 (*cox1*); the apicoplast caseinolytic protease gene (*clpC*); and the nuclear genes elongation factor 2 (*ef2*) and adenylosuccinate lyase (*asl*). The fruit bats (Pteropodidae spp.) were screened with *Hepatocystis-*specific *cytb*-primers (HepF3/HepR3) (Schaer *et al*., 2013). All primers are listed in Supplementary Table S1. All positive PCR products were sequenced with the amplification primers and run on an ABI-373 sequencer. Bat genetic markers comprising the mitochondrial cytochrome b (*cytb*) and the nuclear introns Acyl-CoA oxidase 2, intron 3 (*acox2*), Rogdi-like protein gene, intron 7 (*rogdi*) and Beta-fibrinogen gene, intron 7 (*fgb*) were amplified and sequenced to verify the morphological identifications of the bat hosts (Supplementary Table S1). All DNA sequences were manually edited using the software Geneious Prime 2021.1 (https://www.geneious.com). Ambiguous base calls or missing data were coded with N´s or the corresponding ambiguity code. Sequences were aligned using the Muscle algorithm (Edgar, 2004). Parasite sequences for the analysis of the phylogenetic relationships of *Nycteria* included a total of 3243 nucleotides (nt) (1119 nt of *cytb*, 861 nt of *cox1*, 528 nt of *clpC*, 513 nt of the *ef2-*gene and 222 nt of the *asl-*gene) and for the phylogenetic relationships of *Hepatocystis* included a total of 1938 nucleotides (nt) (531 nt of *cytb*, 993 nt of *cox1* and 513 nt of the *ef2-*gene). Reference sequences were retrieved from GenBank and added to the alignments (all accession numbers are listed in Supplementary Table S2). All individual gene alignments were concatenated, and phylogenetic relationships were evaluated with Bayesian analysis using the taxon *Leucocytozoon* as outgroup. Data were partitioned according to the number of genes and the software PartitionFinder *vs* 2 was used to test different DNA substitution models (Lanfear *et al*., 2017). MrBayes v3.2.7a (Huelsenbeck and Ronquist, 2001) was performed *via* the CIPRES Science Gateway Web Portal V3.3 (Miller *et al*., 2010) with two runs of four chains (three heated, one cold, temperature = 0.03) each for 20 million generations. The first 25% of trees were discarded as burn-in. Mixing and convergence of runs and effective sample size (average ESS > 4000) were assessed with Tracer v1.6 (Rambaut *et al*., 2014). Figures were created with Microsoft PowerPoint and with BioRender.com.

## Results

### Prevalence of haemosporidian infections

A total of 155 bats belonging to five bat families, ten genera and 14 species were investigated. Based on thorough microscopic examinations of thin blood smears (155/155) and PCR screening (153/155), haemosporidian parasites were detected in 28 individuals, corresponding to an overall prevalence of 18.1% (Table 1).

**Table 1.** Investigated bat species and their corresponding haemosporidian parasites

Bat family	Bat species	No. of infected/total individuals	Prevalence in %	Parasite genus
Hipposideridae^a^	*Doryrhina cyclops*	6/10	60.0	*Nycteria*
	*Hipposideros abae*	0/2	0	—
	*Hipposideros curtus*	0/33	0	—
	*Hipposideros fuliginosus*	0/20	0	—
	*Hipposideros ruber*	0/25	0	—
Pteropodidae^b^	*Eidolon helvum*	0/1	0	—
	*Epomops franqueti*	0/15	0	—
	*Epomophorus pusillus*	19/29	65.5	*Hepatocystis*
	*Rousettus aegyptiacus*	0/1	0	—
Rhinolophidae^a^	*Rhinolophus alcyone*	0/2	0	—
	*Rhinolophus landeri*	2/10^c^	20.0	*Nycteria*
Nycteridae^d^	*Nycteris grandis*	1/1^e^	100	*Nycteria*
Vespertilionidae^a^	*Glauconycteris humeralis*	0/1	0	—
	*Laephotis nanus*	0/5	0	—
Total		28/155	18.1	

a Insectivorous bats.

b Frugivorous bats.

c Infection in 1/2 samples was only confirmed by microscopy, no DNA available.

d Mainly insectivorous.

e Infection only confirmed by microscopy, no DNA available.

Haemosporidian parasites were detected in four of the five bat families: Pteropodidae, Hipposideridae, Nycteridae and Rhinolophidae. Morphology of the parasitic blood stages (gametocytes), and molecular analysis identified *Hepatocystis* parasites in *Epomophorus pusillus* (formerly *Micropteropus pusillus*) (Pteropodidae) with a high prevalence of 65.5% (19/29). *Nycteria* parasites were identified in *Doryrhina cyclops* (formerly *Hipposideros cyclops*) (Hipposideridae) with a high prevalence of 60.0% (6/10), in *Rhinolophus* cf. *landeri* (Rhinolophidae) with a lower prevalence of 20.0% (2/10) and a single *Nycteris grandis* (1/1). The bat species that comprised *Nycteria-*infected individuals were exclusively sampled in the forest habitat during the wet season (Supplementary Table S3). Interestingly, none of the *E. pusillus* individuals that were sampled in the forest during the wet season did feature infections with *Hepatocystis* parasites (only one individual that was captured in the dry season was infected). However, all 18 *E. pusillus* that were sampled in the farmland habitat during the wet season were infected with *Hepatocystis* (Supplementary Table S3). Notably, no haemosporidian infection was observed in the savanna zones during our sampling.

**Fig. 2.**
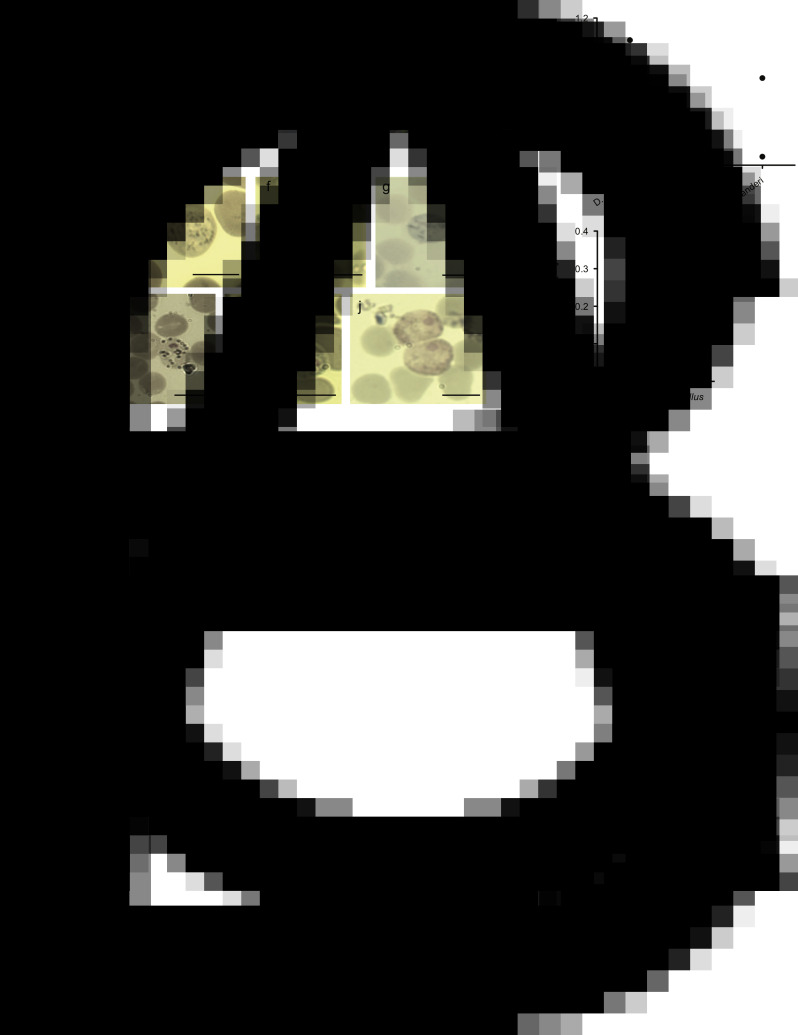
(A) Pictures of bat host species of the study (a) *D. cyclops*; (b) *R.* cf. *landeri*; (c) *E. pusillus*; (B) Representative micrographs of Giemsa-stained thin blood films of mature gametocytes of *Nycteria* parasites: (d, e) gametocytes ex *Doryrhina cyclops*; (f, g) ex *R. landeri*; (h) ex *Nycteris grandis;* (i) representative micrographs of mature macrogametocytes and (j) microgametocytes of *Hepatocystis* parasites of *E. pusillus.* Bars = 5 *μ*m. (C) Parasitaemia per bat individual in percent: (k) parasitaemia values of *Nycteria* and (l) *Hepatocystis* parasitaemia with a mean of 0.11% (± 0.10), maximum values of 0.37% and minimum values of 0.02% in the wet season.

For bats with confirmed haemosporidian infection, parasitaemia values (% of infected erythrocytes) were calculated (Fig. 2C, Table 2, Supplementary Table S4). Mean parasitaemia values in *Hepatocystis*-infected bats (*n* = 19) were 0.11 ± 0.10, with maximum values of 0.37% and minimum values of 0.02% (Table 2). Parasite loads in *Nycteria-*infected *D. cyclops* individuals (*n* = 6) ranged between 0.05 and 1.02% (mean = 0.23% ± 0.39). One *Rhinolophus* sp. individual exhibited 0.71% and the other 0.07% parasitaemia. The single *N. grandis* individual featured a high parasitaemia of 0.21%.

**Table 2.** Parasitaemia values of haemosporidian infection for infected bat families.

Bat host species	M ± s.d. in (%) infected (n)	Max in (%)	Min in (%)
*Doryrhina cyclops*	0.23 ± 0.39 (6)	1.02	0.05
*Epomophorus pusillus*	0.11 ± 0.10 (19)	0.37	0.02
*Rhinolophus* sp.	0.39 ± 0.45 (2)	0.71	0.07
*Nycteris grandis*	0.21 (1)	–	–

M, mean values of parasitemia; s.d., standard deviation.

### Genotyping of infected bat species

The morphological bat species identifications for *E. pusillus, D. cyclops* and *R. landeri* were confirmed with molecular barcoding. A part of the mitochondrial cytochrome *b* (500 bp) was sequenced for four *E. pusillus* individuals, which featured highest nucleotide identity of 99.4% with an *E. pusillus* (*M. pusillus*) reference (KX822887) and 99.4% with an *Epomophorus gambianus* reference sequence (JF728753). This was followed by sequencing 650 bp of *fgb*, which resulted in a nucleotide identity of 100% with the *E. pusillus* reference (JF728439). The nuclear introns *acox2* (500 bp) and *rogdi* (390 bp) were sequenced for three and two individuals of *D. cyclops*, respectively. The *acox2* sequences featured a 100% nucleotide identity with both *Doryrhina* cf. *camerunensis* (e.g. MT149618) and *D. cyclops* (e.g. MT149621). The *rogdi* sequences featured a 98.5% (e.g. FJ85201) nucleotide identity with both *Doryrhina* cf. *camerunensis* (e.g. MT149421) and *D. cyclops* (e.g. MT149423). However, the morphological identification unambiguously identified the individuals of the study as belonging to the species *D. cyclops* (mean forearm length = 65 mm; total length = 109 mm) (Happold and Happold, 2013). A part of the mitochondrial cytochrome *b* (500 bp) was sequenced for one *R. landeri* individual, which featured highest nucleotide identity of 94.1% with a *R. landeri* reference sequence (FJ185201), which could mean, according to the identity scores, that the *R. landeri* samples of the study might belong to another *Rhinolophus* species.

### Morphological and phylogenetic characterization of the haemosporidian parasites

Morphology of gametocyte stages of the *Nycteria* parasites clearly differed between the different host genera (Fig. 2Bd–h). The mature gametocytes of *Nycteria* parasites of *D. cyclops* in the current study resemble the morphology of the *Nycteria* gametocytes described from some rhinolophid hosts (e.g. Krampitz and de Faveaux, 1960; Rosin *et al*., 1978; Schaer *et al*., 2015). The gametocytes feature no apparent nuclear area and a distinction between macro- and microgametocytes was not possible in these samples. The cytoplasm stains light blue with fine haemozoin pigment distributed throughout the parasite cell, which does not fill the entire erythrocyte and its round margin is clearly visible (Fig. 2Bd, e). The gametocytes observed in our samples do slightly also resemble the gametocyte morphology that was reported from the same host species, with the exception that we did not detect distinct nuclei (Lutz *et al*., 2016). The bad quality of the stained blood smears of the *Nycteria* parasites of *R. landeri* did not allow a proper morphological investigation (Fig. 2Bf, g). The mature gametocytes of *Nycteria* parasites of *N. grandis* featured distinct nuclei with dense chromatin. The dark haemozoin pigment distributed in the cytoplasm had a granular appearance (Fig. 2Bh). Morphology of the gametocytes resembled the reported gametocyte morphology from the same host species in Schaer *et al*. (2015), but could not be assigned to known morphospecies.

**Fig. 3.**
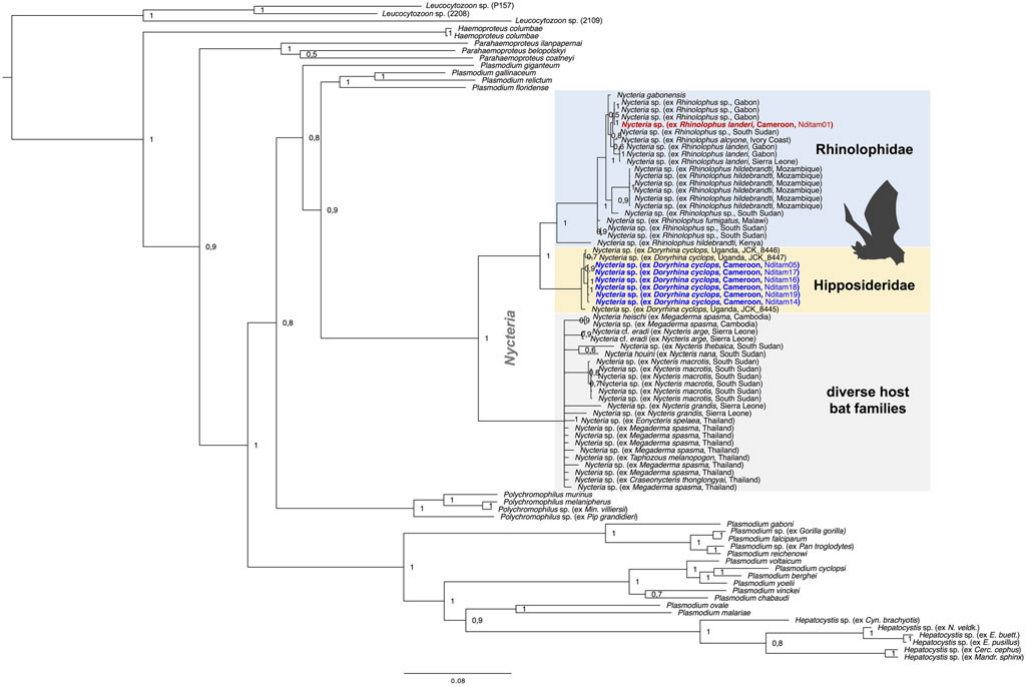
Multi-gene phylogeny of *Nycteria* parasites in the context of the major haemosporidian parasite clades recovered by Bayesian analysis. Posterior probabilities values are given. The analysis is based on the concatenated alignment (total of 3243 bp) of the mitochondrial genes *cytb* (1119 bp) and *cox1* (861 bp), the apicoplast marker *clpC* (528 bp) and the nuclear *ef2-*gene (513 bp) and *asl-*gene (222 bp). Placement of *Nycteria* parasites as sister to a clade that contains the lizard and bird *Plasmodium* species. The monophyletic *Nycteria* clade contains representative reference sequences of *Nycteria* parasites of diverse bat families from Asia and Africa. The samples of the current study are highlighted in bold blue (ex *Doryrhina cyclops*) and bold red (ex *R. landeri*). *Nycteria* parasites of African *Rhinolophus* host species (blue clade) and the African hipposiderid *Doryrhina* hosts (yellow clade) group in their own host bat family specific clades. The grey clade contains *Nycteria* sequences from different host bat families in Asia (Pteropodidae, Megadermatidae, Nycteridae and Craseonycteridae).

Phylogenetic analyses were performed to assess the phylogenetic diversity, and possible geographic and/or host-specificity patterns of haemosporidian parasites (*Hepatocystis* and *Nycteria)* from Cameroon. The multi-gene phylogeny of *Nycteria* parasites (two mitochondrial, two nuclear and one apicoplast gene) recovered the *Nycteria* clade as sister to a clade that contains the lizard and bird *Plasmodium* species as shown before (Fig. 3) (e.g. Schaer *et al*., 2015). However, other studies, including the most comprehensive haemosporidian phylogeny, which was based on several nuclear markers, placed *Nycteria* as basal clade to the mammalian *Plasmodium/Hepatocystis* parasites (e.g. Schaer *et al*., 2013; Galen *et al*., 2018). Representative reference sequences of *Nycteria* parasites of diverse bat families from Asia and Africa were included in the analysis. The *Nycteria* sequences of *D. cyclops* of the current study are grouped closely with *Nycteria* parasites of *D. cyclops* from Uganda in one monophyletic clade (Fig. 3). *Nycteria* parasites of African *Rhinolophus* host species including the parasite sequence of *Rhinolophus* sp. from Cameroon also group in their own monophyletic host bat family-specific *Nycteria* clade that contains sequences from the two species *Nycteria* cf. *gabonensis* and *Nycteria* cf. *congolensis* (Schaer *et al*., 2015; Rosskopf *et al*., 2019). Therefore, the parasites from *R.* cf. *landeri* of the current study could represent either *N.* cf. *gabonensis* or *N.* cf. *congolensis.* The third *Nycteria* subclade contains parasite sequences from different host bat families in Asia (Pteropodidae, Megadermatidae, Nycteridae and Craseonycteridae). The placement of *Nycteria* of the *Nycteris* hosts within this clade is uncertain as no reference sequences are available for *cytb, cox1, asl* and *ef2* for these parasites and thus the concatenated sequences contain a large amount of missing data. Despite using different protocols to amplify and sequence the apicoplast *clpC* marker, no *clpC* sequences were successfully generated for the *Nycteria* parasites of the study. However, the phylogenetic analysis was run with a concatenated dataset that included *clpC* from reference sequences to analyse the placement of *Nycteria* parasites from *Nycteris* hosts (Fig. 3). We also ran a dataset without the *clpC* data, which resulted in the same tree topology (Supplementary Fig. S1).

**Fig. 4.**
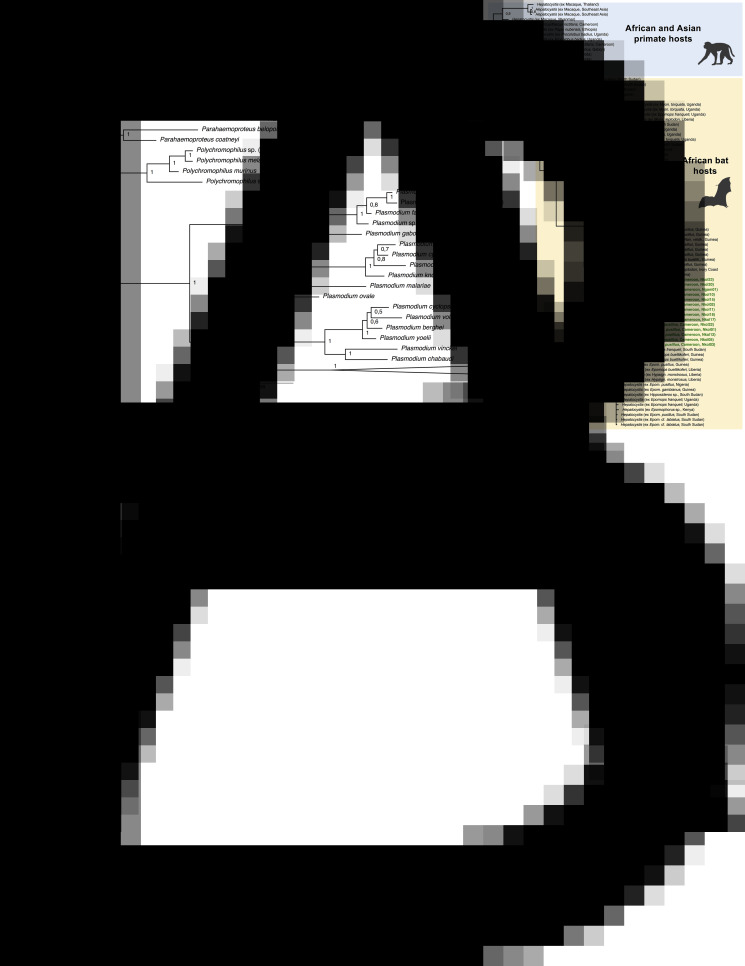
(A) Phylogenetic analysis of *Hepatocystis* parasites in the context of some major haemosporidian clades recovered by Bayesian analysis. Posterior probabilities are given. The analysis is based on the concatenated alignment (total of 1983 bp) of the mitochondrial cytochrome b (531 bp) and cytochrome oxidase 1 (993 bp) and the nuclear elongation factor 2 (513 bp). (B) Placement of *Hepatocystis* as collapsed clade as sister clade to the mammalian *Plasmodium* (*Plasmodium*) and *Plasmodium* (*Vinckeia*) clade. (B) The uncollapsed clade contains representative reference sequences of *Hepatocystis* of primate hosts from Asia and Africa as well as African bat hosts. The samples of the current study are highlighted in bold green. Sequences of the *Hepatocystis* parasites from *E. pusillus* bats in Cameroon closely group within the *Hepatocystis* parasite clades of epauletted fruit bat species from different African countries and no host species specificity is apparent.

The morphology of the gametocyte blood stages of the *Hepatocystis* parasites of *E. pusillus* in Cameroon corresponds to the descriptions of *Hepatocystis epomophori* parasites of epauletted fruit bats from other African countries (Garnham, 1966; Schaer *et al*., 2013, 2017) (Fig. 2B). The sexually dimorphic macro- and microgametocytes were distinguished after Giemsa staining, the macrogametocytes stained blue, with a red staining nucleus of smaller size (in comparison with the nucleus of the microgametocyte) (Fig. 2Bi). The microgametocytes featured pale brown cytoplasm with evenly distributed haemozoin except for the nuclear zone (Fig. 2Bj). Bayesian analyses confirmed *Hepatocystis* as a monophyletic clade with high support (posterior probability of 1) and as sister clade to a clade that comprises the *Plasmodium* (*Plasmodium*) and *Plasmodium* (*Vinckeia*) parasites (Fig. 4). The analysis also recovered all major clades [*Leucocytozoon, Haemoproteus, Parahaemoproteus, Polychromophilus, Plasmodium* (*Plasmodium*), *Plasmodium* (*Vinckeia*), *Plasmodium* (*Laverania*)] as monophyletic. The *Hepatocystis* clade is comprised of two main subclades, the first including all parasites of Asian and African primates, the second main group contains African chiropteran *Hepatocystis* parasites including the *Hepatocystis* parasites of the study (Fig. 4B). Within the African bat *Hepatocystis* clade, parasite sequences do not cluster in host genus or species-specific clades but represent several close related taxa or cryptic species as shown before (Schaer *et al*., 2017). Further, sequences from different countries and locations in both West Africa and Central-/East Africa fall in several different places across the tree, showing no pattern of clustering according to countries. The sequences of *E. pusillus* from Central Cameroon group closely together, confirming high sequence similarities between each other. However, together they group with West and Central-/East African *Hepatocystis* parasites of *E. pusillus* and other epauletted fruit bat species on a polytomous branch, with whom they also share very close phylogenetic relationships.

## Discussion

This study provides the first information on haemosporidian parasite infections in bats in Cameroon. Bats are one of the most species-rich groups of mammals in the Central region of Cameroon in the equatorial tropical area, that features different habitat types (forest, savanna and cultivated farms) (Atagana *et al*., 2018; Mbeng *et al*., 2019). Haemosporidian parasite infections were identified in 28 out of 155 investigated bats, belonging to four different bat families. *Hepatocystis* parasites were discovered in *E. pusillus* with a high prevalence of 65.5%, which is in line with previous reports from *Hepatocystis* infections in epauletted fruit bats in other African countries (e.g. Schaer *et al*., 2013, 2017; Lutz *et al*., 2016; Boundenga *et al*., 2018; Atama *et al*., 2019). No infections were detected in the other sampled epauletted fruit bat species, *E. franqueti,* which has also been recorded as a host of *Hepatocystis* in previous studies (e.g. Lutz *et al*., 2016; Schaer *et al*., 2017). All *E. franqueti* were captured in the forest habitat in the wet season, along with nine *E. pusillus* that were also uninfected. Strikingly, all 18 *E. pusillus* individuals that were investigated in the cultured farmland habitat during the wet season were infected with *Hepatocystis*, which points to high transmission rates in the cultured habitat due to a potentially high abundance of the invertebrate vector in the farmland in contrast to the forest habitat.

The parasites of *E. pusillus* from Central Cameroon might belong to one of the diverse lineages of the *Hepatocystis epomophori* species complex as the parasites feature a close relationship with *Hepatocystis* parasites of diverse fruit bat host species from West and Central-/East Africa, which comprise parasites that have tentatively been assigned to the morphospecies *H. epomophori* (Schaer *et al*., 2017). Diverse assemblages of cryptic species have also been detected in *Hepatocystis* parasites of primate hosts (Thurber *et al*., 2013).

*Nycteria* parasites were discovered in *D. cyclops* with a high prevalence of 60.0%. This is only the second time that this parasite genus has been reported from this host species and again, with a high prevalence (Lutz *et al*., reported three out of three infected *D. cyclops* in Uganda) (Lutz *et al*., 2016). Despite the geographical distance, our phylogenetic analysis recovered a very close relationship between the *Nycteria* parasites of *D. cyclops* from Uganda and Cameroon, with no nucleotide differences in the partial *cytb* sequences and less than 1% sequence divergence for the partial *cox1* gene sequences. No *Nycteria* morphospecies has been described from *D. cyclops* and based on the distinct monophyletic phylogenetic relationship of the parasite sequences of *D. cyclops,* these parasites might represent a distinct/new *Nycteria* species, which is host species specific to *D. cyclops* with a corresponding distribution range in Africa. Interestingly, the bat species *D. cyclops* has also been identified as host species of another haemosporidian species, *Plasmodium cyclopsi* (Landau and Chabaud, 1978; Schaer *et al*., 2013). In both cases, *Nycteria* and *P. cyclopsi*, the identity of the dipteran vector species and even family is unknown. *Nycteria* parasites were also identified in one of two investigated *Rhinolophus* cf. *landeri* individuals from Cameroon, a host species that has been repeatedly reported as host of *Nycteria* parasites in several other African countries, which renders *R.* cf. *landeri* as common host of *Nycteria* (e.g. Krampitz and de Faveaux, 1960; Schaer *et al*., 2015; Rosskopf *et al*., 2019). The study confirms a specificity of *Nycteria* parasites in regard to their bat host families for the parasites of rhinolophid and hipposiderid hosts (e.g. Schaer *et al*., 2015; Lutz *et al*., 2016). A previous study found *Nycteria* parasites of the *Nycteris* hosts also to be monophyletic (Schaer *et al*., 2015). Our phylogenetic analysis recovered the *Nycteria* parasites of African *Nycteris* hosts within a clade that comprises parasite sequences from the different host bat families Pteropodidae, Megadermatidae, Emballonuridae and Craseonycteridae from Asia. However, this placement must be considered with caution, as for most of the Asian parasites the molecular data are limited to short reference sequences of the *cytb* gene (e.g. Arnuphapprasert *et al*., 2020). Further, for most parasites of *Nycteris* hosts, reference sequences for *cytb* and *cox1* (as well as for *asl* and *ef2*) are missing and thus the concatenated sequences contain a large amount of missing data (Schaer *et al*., 2015). Additionally, a previous study has shown that the genes in the mitochondrial genome of *Nycteria* parasites of *Nycteris* hosts are rearranged and thus the mitochondrial genome differs from that of other *Nycteria* parasites (of other host genera) and to that of all other haemosporidian genera (Karadjian *et al*., 2016).

In conclusion, our survey of haemosporidian parasites in bats in Central Cameroon shows that *Hepatocystis* infections in African epauletted fruit bats varies among bat host species and also between populations of the same bat species in different geographic locations. A high prevalence was confirmed in *E. pusillus* on the one hand, but infections were lacking in *E. franqueti* on the other hand. Habitat type and seasonality may play a role in transmission of *Hepatocystis* parasites on a local scale. This study also adds important information on the distribution and host specificity of the neglected haemosporidian genus *Nycteria.*

Cameroon is known as ‘Africa in miniature’ as it mirrors the continent´s diverse habitats and biodiversity. Future systematic sampling and longitudinal studies across the country with its diverse bat fauna are needed to assess the full diversity of haemosporidian parasites in bats in Cameroon, to study the transmission biology of haemosporidian parasites, their effects on the bat hosts and to identify their insect hosts.

## Supplementary material

For supplementary material accompanying this paper visit https://doi.org/10.1017/S0031182021001542. click here to view supplementary material

## Data

All sequences of the study are available at GenBank (NCBI) with the accession numbers MZ451636-MZ451644, MZ460913-MZ460953, MZ460988-MZ460992.
