# Supplementary material for: Hepatocystis and Nycteria (Haemosporida) parasite infections of bats in the Central Region of Cameroon
Source: Parasitology. 2021 Sep 6;149(1):51–8. doi: 10.1017/S0031182021001542 (PMC8862134; doi:10.1017/S0031182021001542)
Supplement: Supplementary file 1 [file S0031182021001542sup001.docx]

*Hepatocystis* and *Nycteria* (Haemosporida) parasite infections of bats in the Central Region of Cameroon

Tsague K.J.^1,2^, Bakwo Fils E.M.^1^, Atagana J.P.^1^, Dongue NV^2^, Mbeng D.W.^1^, Schaer J.^3,*,§^, Tchuinkam T.^2,*,§^

**Supplemental Material**

- **Supplementary Figure S1**. Phylogeny of *Nycteria* parasites (concatenated dataset of *cytb, cox1, ef2, asl*)
- **Supplementary Table S1**. Nucleotide primers used in this study
- **Supplementary Table S2**. GenBank accession numbers
- **Supplemental Table S3**: Overview of number of investigated bat individuals and infections per sampling sites and season and across sex and age groups
- **Supplemental Table S4**: Overview of infected bat individuals of the study, sampling habitat, sampling season and parasitemia

**Supplemental Figure S1:** Multi-gene phylogeny of *Nycteria* parasites in the context of the major haemosporidian parasite clades recovered by Bayesian analysis. Posterior probabilities values are given. The analysis is based on the concatenated alignment (total of 2715bp) of the mitochondrial genes *cytb* (1119 bp) and *cox1* (861 bp), the nuclear *ef2-*gene (513 bp) and *asl-*gene (222 bp). Placement of *Nycteria* parasites as sister to a clade that contains the lizard and bird *Plasmodium* species. The monophyletic *Nycteria* clade contains representative reference sequences of *Nycteria* parasites of diverse bat families from Asia and Africa. The samples of the current study are highlighted in blue (ex *Doryrhina cyclops*) and red (ex *Rhinolophus landeri*). *Nycteria* parasites of African *Rhinolophus* host species and the African hipposiderid *Doryrhina* hosts group in their own host bat family specific clades. The grey clade contains *Nycteria* sequences from different host bat families in Asia (Pteropodidae, Megadermatidae, Nycteridae and Craseonycteridae).

**Supplemental Table S1:** Nucleotide primers used in this study

| **Gene** | **Primer name** | **Sequence (5´- 3´)** | **Reference** |
| --- | --- | --- | --- |
| ***cytb***  (haemosporidian parasites) | Hep-F3 | CTTACCTTGGGGACAAATGAGTTATT | Schaer et al., 2013 |
|  | Hep-R3 | CTCTAGCACCAAATGTCATTTTAAATTG | Schaer et al., 2013 |
|  | DW2 | TAATGCCTAGACGTATTCCTGATTATCCAG | Perkins and Schall, 2002 |
|  | DW4 | TGTTTGCTTGGGAGCTGTAATCATAATGTG | Perkins and Schall, 2002 |
|  | 3932-F | GGGTTATGTATTACCTTGGGGTC | Perkins and Schall, 2002 |
|  | 3932-R | GACCCCAAGGTAATACATAACCC | Perkins and Schall, 2002 |
| ***cox1***  (haemosporidian parasites) | Cox1-F | CTATTTATGGTTTTCATTTTTATTTGGTA | Martinsen et al., 2008 |
|  | Cox1-R | AGGAATACGTCTAGGCATTACATTAAATCC | Martinsen et al., 2008 |
|  | Cox-in-F | ATGATATTTACARTTCAYGGWATTATTATG | Martinsen et al., 2008 |
|  | Cox-in-R | GTATTTTCTCGTAATGTTTTACCAAAGAA | Martinsen et al., 2008 |
|  | Cox-mid-F | TTATTCTGGTTTTTTGGTCATCCAG | Martinsen et al., 2008 |
|  | Cox-mid-R | CTGGATGACCAAAAAACCAGAATAA | Martinsen et al., 2008 |
| ***clpc***  (haemosporidian parasites) | Clpc-out-F | AAACTGAATTAGCAAAAATATTA | Martinsen et al., 2008 |
|  | Clpc-out-R | CGWGCWCCATATAAAGGAT | Martinsen et al., 2008 |
|  | Clpc-in-F | GATTTGATATGAGTGAATATATGG | Martinsen et al., 2008 |
|  | Clpc-in-R | CCATATAAAGGATTATAWG | Martinsen et al., 2008 |
|  | clpCF | GTTGGATTTTATGTGGDCCTAGTGG | Falk et al., 2015 |
|  | clpCR | AAWGGACGWGCWCCATATAAAGG | Falk et al., 2015 |
|  | clpCFin | TCTATTTCTAGATTAATAGG | Falk et al., 2015 |
|  | clpCRin | AAGGATTATAAGATAATTTAG | Falk et al., 2015 |
| ***ef2***  (haemosporidian parasites) | EF2-F | GTTCGTGAGATCATGAACAAAAC | Schaer et al., 2013 |
|  | EF2-R | CCTTGTAAACCAGAACCAAA | Schaer et al., 2013 |
|  | EF2F | CATGGAAAATCAACATTAACAGATTCT | Falk et al., 2015 |
|  | EF2R | CAGGATATACTTGAATATCACCCAT | Falk et al., 2015 |
|  | EF2Fin | AGACAAGATGAACAAGAAAGATGT | Falk et al., 2015 |
|  | EF2Rin | TCACCCATTAATTTATCTGTGTATGT | Falk et al., 2015 |
| ***asl***  (haemosporidian parasites) | ASL-F | GSKAARTTTAATGGKGCTGTWGG | Martinsen et al., 2008 |
|  | ASL-R | GGATTAAYTTTATGAGGCATTG | Martinsen et al., 2008 |
|  | ASL-in-F | GCTGATMAAAATRTTGATTGG | Martinsen et al., 2008 |
|  | ASL-in-R | GAGGCATTGTACTACTWCC | Martinsen et al., 2008 |
| ***cytb***  (bat hosts) | L14724 | CGAAGCTTGATATGAAAAACCATCGTTG | Päabo 1989 |
|  | H15915 | GGAATTCATCTCTCCGGTTTACAAGAC | Irwin et al. 1991 |
| ***fgb***  (bat hosts) | FGB-F | CCACAACRGCATGTTCTTCAGCAC | Hassanin, 2007 |
|  | FGB-R | GTATCTGCCATTTGGATTGGCTGC | Hassanin, 2007 |
| ***acox2***  (bat hosts) | ACOX2-F1 | CCTSGGCTCDGAGGAGCAGAT | Salicini, 2011 |
|  | ACOX2-R1 | GGGCTGTGHAYCACAAACTCCT | Salicini, 2011 |
| ***rogdi***  (bat hosts) | ROGDI-F1 | CTGATGGAYGCYGTGATGCTGCA | Salicini, 2011 |
|  | ROGDI-R1 | CACGGTGAGGCASAGCTTGTTGA | Salicini, 2011 |

*cytb*, cytochrome b; *cox1*, cytochrome oxidase I; *clpc*, apicoplast caseinolytic protease; *ef2*, nuclear elongation factor 2; *asl,* adenylosuccinate lyase; Nuclear introns for bat genotyping: *acox*2 (Acyl-CoA oxidase 2, intron 3); *rogdi* (Rogdi-like protein gene, intron 7); *fgb* (Beta-fibrinogen gene, intron 7)

| **References**  Falk, B.G., Glor, R.E., Perkins, S.L., 2015. Clonal reproduction shapes evolution in the lizard malaria parasite *Plasmodium floridense*. Evolution. 69, 1584-1596.  Hassanin, A., Ropiquet, A. 2007. Resolving a zoological mystery: the Kouprey is a real species, Proc. R. Soc. Lond. B. 274 (2007) 2849–2855.  Irwin, D.M., Kocher, T.D., Wilson, A.C., 1991. Evolution of the Cytochrome *b* gene of mammals. *J. Mol. Evol.* 32, 128 – 144.  Martinsen, E.S., Perkins, S.L., Schall, J., 2008. A three-genome phylogeny of malaria parasites (*Plasmodium* and closely related genera): evolution of life-history traits and host switches. Mol. Phylogenet. Evol. 47, 261–273.  Paäbo, S., 1989. Ancient DNA extraction, characterization, molecular cloning and enzymatic amplification. PNAS 86, 1939–1943.  Perkins, S.L., Schall, J., 2002. A molecular phylogeny of malarial parasites recovered from cytochrome *b* gene sequences. J. Parasitol. 88, 972–978.  Salicini, I., Ibanez, C., Juste, J., 2011. Multilocus phylogeny and species delimitation within theNatterer's bat species complex in the Western Palearctic. Mol. Phylogenet. Evol. 61, 888–898.  Schaer, J., Perkins, S.L., Decher, J., Leendertz, F.H., Fahr, J., Weber, N., Matuschewski, K., 2013. High diversity of West African bat malaria parasites and a tight link with rodent *Plasmodium* taxa. Proc. Natl. Acad. Sci. USA. 110, 17415-17419.  **Supplemental Table S2:** GenBank accession numbers for phylogenetic analysis (samples from this study highlighted in bold)   \| **Parasite (host group)** \| **Sample (host species)** \| ***cytb*** \| ***cox1*** \| ***clpc*** \| ***ef2*** \| ***asl*** \| \| --- \| --- \| --- \| --- \| --- \| --- \| --- \| \| *Leucocytozoon* (Aves) \| *Leucocytozoon* sp. (2109) \| EU254518 \| EU254563 \| EU254609 \| — \| EU254663 \| \| *Leucocytozoon* sp. (2208) \| EU254520 \| EU254565 \| EU254611 \| — \| EU254665 \| \| *Leucocytozoon* sp. (P157) \| EU254519 \| EU254564 \| EU254610 \| — \| EU254664 \| \| *Haemoproteus* (Aves) \| *HHaemoproteus columbae* (2111) \| EU254548 \| — \| EU254642 \| — \| EU254699 \| \| *Haemoproteus columbae* (2146) \| EU254553 \| — \| EU254652 \| — \| EU254707 \| \| *Haemoproteus columbae* \| FJ168562 \| FJ168562 \| — \| — \| — \| \| *Haemoproteus columbae* \| NC012448 \| NC012448 \| — \| — \| — \| \| *Haemoproteus columbae* \| KY653761 \| KY653761 \| — \|  \| — \| \| *Parahaemo-proteus* (Aves) \| *Parahaemoproteus belopolskyi* \| DQ451408 \| EU254603 \| EU254657 \| — \| EU254710 \| \| *Parahaemoproteus coatneyi* \| EU254550 \| EU254595 \| EU254648 \| — \| EU254704 \| \| *Parahaemoproteus ilanpapernai* \| DQ451424 \| EU254591 \| EU254643 \| — \| EU254700 \| \| *Plasmodium* (Aves, Squamata) \| *Plasmodium floridense* \| EF079654 \| EF079654 \| EU254620 \| JN187874 \| EU254675 \| \| *Plasmodium gallinaceum* \| AY099029 \| LN835294 \| AB649424 \| — \| AF262053 \| \| *Plasmodium giganteum* \| AY099053 \| EU254577 \| EU254624 \| — \| LN483056 \| \| *Plasmodium mexicanum* \| EF079653 \| EF079653 \| EU254619 \| — \| EU254674 \| \| *Plasmodium relictum* \| AY733090 \| AY733090 \| EU254633 \| — \| XM_028680302 \| \| *Plasmodium* (Primates) \| *Plasmodium falciparum* \| DQ642845 \| M76611 \| DQ642846 \| DQ642846 \| KP050472 \| \| *Plasmodium gaboni* \| FJ895307 \| FJ895307 \| HQ842630 \| — \| — \| \| *Plasmodium malariae* \| AF069624 \| AB489193 \| AF348342 \| — \| XM_029003257 \| \| *Plasmodium ovale* \| AF069625 \| JF894415 \| AY634623 \| — \| KP050468 \| \| *Plasmodium reichenowi* \| AJ251941 \| AJ251941 \| EU560464 \| — \| — \| \| *Plasmodium* sp. (ex *P. troglodytes*) \| HM235391 \| HM235391 \| HM235147 \| — \| — \| \| *Plasmodium* sp. (ex *G. gorilla*) \| HM235288 \| HM235308 \| HM235163 \| — \| — \| \| *Plasmodium cynomolgi* \| AF069616 \| MN372343 \| AB471873 \| — \| — \| \| *Plasmodium inui* \| AF069617 \| AB354572 \| AB471879 \| — \| — \| \| *Plasmodium knowlesi* \| AF069621 \| EU880499 \| AF348341 \| XM002260326 \| AF069621 \| \|  \| *Plasmodium vivax* \| KY923424 \| KY92342 \| — \| XM_001615828 \| — \| \| *Plasmodium* (Rodentia) \| *Plasmodium berghei* \| DQ414645 \| DQ414589 \| DQ417612 \| — \| AF262049 \| \| *Plasmodium chabaudi* \| DQ414649 \| DQ414593 \| DQ417616 \| XM_736543 \| EU254668 \| \| *Plasmodium vinckei* \| DQ414651 \| DQ414596 \| DQ417619 \| — \| EU254667 \| \| *Plasmodium yoelii* \| AY099051 \| DQ414605 \| DQ417628 \| LM993667 \| EU254666 \| \| *Plasmodium* (Chiroptera) \| *Plasmodium cyclopsi* \| KF159710 \| KF159788 \| KF159635 \| KF159729 \| KF159653 \| \| *Plasmodium voltaicum* \| KF159671 \| KF159792 \| KF159648 \| — \| KF159654 \| \| *Polychromophilus* (Chiroptera) \| *Polychrom.* sp. (ex *Mini. villiersii*) \| KF159681 \| KF159796 \| KF159642 \| KF159731 \| — \| \| *Polychromophilus melanipherus* \| JN990709 \| — \| JN990721 \| — \| JN990726 \| \| *Polychromophilus murinus* \| HM055583 \| — \| JN990723 \| — \| JN990725 \| \| *Polychrom.* sp. (ex *P. grandidieri*) \| KF159714 \| KF159797 \| KF159639 \| KF159742 \| — \| \| *Hepatocystis* (Primates) \| *H.* sp. (ex *Cerc. cephus*) \| JF923760 \| — \| — \| — \| — \| \| *H.* sp. (ex *Cerc. cephus*) \| JF923758 \| — \| — \| — \| — \| \| *H.* sp. (ex *Cerc. nictitans*) \| JQ070956 \| — \| — \| — \| — \| \| *H.* sp. (ex *Cerc. nictitans* \| JQ070816 \| — \| — \| — \| — \| \| *H.* (ex Macaque, Myanmar) \| HQ605040 \| — \| — \| — \| — \| \| *H.* (ex Macaque, Southeast Asia) \| GU929945 \| — \| — \| — \| — \| \| *H.* (ex Macaque, Southeast Asia) \| GU929944 \| — \| — \| — \| — \| \| *H.* (ex Macaque, Thailand) \| EU400409 \| — \| — \| — \| — \| \| *H.* sp. (ex *M. sphinx*) \| JF923759 \| — \| — \| — \| — \| \| *H.* (ex *Miopithecus talapoin*, Gabon) \| JF923757 \| — \| — \| — \| — \| \| *H.* (ex *Papio nubensis*, Ethiopia) \| AF069626 \| — \| — \| — \| — \| \| *H.* (ex *Procolobus badius*, Uganda) \| KC262797 \| — \| — \| — \| — \| \| *H.* (ex *Procolobus badius*, Uganda) \| KC262824 \| — \| — \| — \| — \| \| *H.* (ex *Procolobus badius*, Uganda) \| KC262867 \| — \| — \| — \| — \| \| *H.* (ex *Procolobus badius*, Uganda) \| GU945305 \| — \| — \| — \| — \| \| *Hepatocystis* (Chiroptera) \| *H.* sp. (ex *C. brachyotis*) \| EU254526 \| EU254569 \| EU254616 \| — \| EU254671 \| \| *H.* sp. (ex *E. gambianus,* Guinea) \| KF159695 \| — \| — \| — \| — \| \| *H.* sp. (ex *E. pusillus,* Guinea) \| KF159683 \| KF159801 \| KF159623 \| KF159744 \| — \| \| *H.* sp. (ex *E. pusillus,* Guinea) \| KF159680 \| KF159778 \| KF159641 \| KF159723 \| — \| \| *H.* sp. (ex *E. pusillus,* Guinea) \| KF159693 \| KF159773 \| KF159645 \| KF159746 \| — \| \| *H.* sp. (ex *E. pusillus,* Guinea) \| KF159704 \| KF159775 \| KF159618 \| KF159753 \| — \| \| *H.* sp. (ex *E. pusillus,* Guinea) \| KF159683 \| KF159801 \| KF159623 \| KF159744 \| — \| \| ***H.* sp. (ex *E. pusillus*, Cameroon), Nkol02** \| **MZ460915** \| — \| — \| — \| — \| \| ***H.* sp. (ex *E. pusillus*, Cameroon), Nkol10** \| **MZ460918** \| — \| — \| — \| — \| \| ***H.* sp. (ex *E. pusillus*, Cameroon), Nkol12** \| **MZ460920** \| **MZ460952** \| — \| — \| — \| \| ***H.* sp. (ex *E. pusillus*, Cameroon), Nkol15** \| **MZ460921** \| **—** \| — \| — \| — \| \| ***H.* sp. (ex *E. pusillus*, Cameroon), Nkol17** \| **MZ460922** \| **—** \| — \| — \| — \| \| ***H.* sp. (ex *E. pusillus*, Cameroon), Nkol18** \| **MZ460923** \| **—** \| — \| — \| — \| \| ***H.* sp. (ex *E. pusillus*, Cameroon), Ngambe01** \| **MZ460914** \| **MZ460949** \| — \| — \| — \| \| ***H.* sp. (ex *E. pusillus*, Cameroon), Nkol01** \| **MZ460913** \| **MZ460950** \| — \| — \| — \| \| ***H.* sp. (ex *E. pusillus*, Cameroon), Nkol03** \| **MZ460916** \| — \| — \| **MZ460927** \| — \| \| ***H.* sp. (ex *E. pusillus*, Cameroon), Nkol05** \| **MZ460917** \| **MZ460951** \| — \| **MZ460928** \| — \| \| ***H.* sp. (ex *E. pusillus*, Cameroon), Nkol11** \| **MZ460919** \| — \| — \| — \| — \| \| ***H.* sp. (ex *E. pusillus*, Cameroon), Nkol20** \| **MZ460924** \| **MZ460953** \| — \| — \| — \| \| ***H.* sp. (ex *E. pusillus*, Cameroon), Nkol22** \| **MZ460925** \| **MZ460948** \| — \| — \| — \| \| ***H.* sp. (ex *E. pusillus*, Cameroon), Nkol23** \| **MZ460926** \| **MZ460947** \| — \| — \| — \| \| *H.* sp. (ex *E. pusillus,* Nigeria) \| MK634490 \| — \| MK634484 \| MK634512 \| — \| \| *H.* sp. (ex *E. pusillus,* Nigeria) \| MK634507 \| — \| MK634486 \| MK634523 \| — \| \| *H.* sp. (ex *E. pusillus,* Nigeria) \| MK634505 \| — \| MK634485 \| MK634521 \| — \| \| *H.* sp. (ex *E. pusillus,* Nigeria) \| MK634501 \| — \| — \| MK634517 \| — \| \| *H.* sp. (ex *E. pusillus,* Nigeria) \| MK634496 \| — \| — \| MK634515 \| — \| \| *H.* sp. (ex *E. pusillus,* Nigeria) \| MK634503 \| — \| — \| MK634519 \| — \| \| *H.* sp. (ex *E. pusillus,* Nigeria) \| MK634487 \| — \| — \| MK634509 \| — \| \| *H.* sp. (ex *E. pusillus,* Nigeria) \| MK634497 \| — \| — \| MK634516 \| — \| \| *H.* sp. (ex *E. pusillus,* Nigeria) \| MK634489 \| — \| — \| MK634511 \| — \| \| *H.* sp. (ex *E. pusillus,* Nigeria) \| MK634506 \| — \| — \| MK634522 \| — \| \| *H.* sp. (ex *E. pusillus,* Nigeria) \| MK634488 \| — \| — \| MK634510 \| — \| \| *H.* sp. (ex *E. pusillus,* SoSu) \| KY753527 \| KY753543 \| KY753567 \| KY753591 \| — \| \| *H.* sp. (ex *E. pusillus,* SoSu) \| KY753525 \| KY753541 \| KY753565 \| KY753589 \| — \| \| *H.* sp. (ex *Epom.* sp*.,* Kenya) \| KY753518 \| KY753536 \| KY753559 \| KY753583 \| — \| \| *H.* sp. (ex *Epom.* sp*.,* Kenya) \| KY753519 \| — \| KY753560 \| KY753584 \| — \| \| *H.* sp. (ex *Epom.* sp*.,* Nigeria) \| MK634504 \| — \| — \| MK634520 \| — \| \| *H.* sp. (ex *Epom.* sp*.,* SoSu) \| KY753506 \| KY753530 \| KY753547 \| KY753571 \| — \| \| *H.* sp. (ex *Epom.* sp*.,* SoSu) \| KY753507 \| KY753531 \| KY753531 \| KY753572 \| — \| \| *H.* sp. (ex *Epom.* ) \| KY753513 \| KY753535 \| KY753554 \| KY753578 \| — \| \| *H.* sp. (ex *Epom.* ) \| KY753510 \| KY753534 \| KY753551 \| KY753575 \| — \| \| *H.* sp. (ex *Epom.* sp*.,* SoSu) \| KY753516 \| — \| KY753557 \| KY753581 \| — \| \| *H.* sp. (ex *E. buettikoferi*, Guinea) \| KF159706 \| KF159779 \| KF159612 \| KF159757 \| — \| \| *H.* sp. (ex *E. buettikoferi*, Guinea) \| KF159703 \| KF159790 \| — \| — \| — \| \| *H.* sp. (ex *E. buettikoferi*, Liberia) \| — \| — \| — \| KF159741 \| — \| \| *H.* sp. (ex *E. franqueti*, Uganda) \| KT750344 \| KT750505 \| KT750688 \| — \| — \| \| *H.* sp. (ex *E. franqueti*, Uganda) \| KT750356 \| KT750527 \| KT750667 \| — \| — \| \| *H.* sp. (ex *Hipposideros* sp.*,* Guinea) \| KY753520 \| — \| — \| — \| — \| \| *H.* sp. (ex *H. monstrosus,* Liberia) \| KF159689 \| KF159799 \| — \| KF159734 \| — \| \| *H.* sp. (ex *H. monstrosus,* Liberia) \| KF159712 \| — \| KF159647 \| — \| — \| \| *H.* sp. (ex *H. monstrosus,* SoSu) \| KY753521 \| KY753537 \| KY753561 \| KY753585 \| — \| \| *H.* sp. (ex *M. torquata,* Uganda) \| KT750356 \| KT750527 \| KT750667 \| — \| — \| \| *H.* sp. (ex *M. torquata,* Uganda) \| KT750357 \| KT750521 \| KT750673 \| — \| — \| \| *H.* sp. (ex *M. torquata,* Uganda) \| KT750342 \| KT750534 \| KT750659 \| — \| — \| \| *H.* sp. (ex *N. veldkampii,* Guinea) \| KF159698 \| KF159786 \| KF159631 \| KF159749 \| — \| \| *H.* sp. (ex *N. veldkampii,* Guinea) \| EU254528 \| EU254571 \| EU254618 \| — \| — \| \| *H.* sp. (ex *N. veldkampii,* Liberia) \| KF159698 \| KF159786 \| KF159631 \| KF159749 \| EU254671 \| \| *H.* sp. (ex *R. aegyptiacus,* Nigeria) \| MK634508 \| — \| MK634483 \| MK634524 \|  \| \| *Nycteria* (Chiroptera) \| *N.* sp. (ex *C. thonglongyai,* Thailand) \| MT136164 \| — \| — \| — \| — \| \| *N.* sp. (ex *E. spelaea,* Thailand) \| MT136166 \| — \| — \| — \| — \| \| *N.* sp. (ex *M. spasma,* Cambodia) \| EF179355 \| — \| — \| — \| — \| \| *N. heischi* (ex *M. spasma,* Cambodia) \| KX090648 \| KX090648 \| — \| — \| — \| \| *N.* sp. (ex *M. spasma,* Thailand) \| MT136157 \| — \| — \| — \| — \| \| *N.* sp. (ex *M. spasma,* Thailand) \| MT136158 \| — \| — \| — \| — \| \| *N.* sp. (ex *M. spasma,* Thailand) \| MT136159 \| — \| — \| — \| — \| \| *N.* sp. (ex *M. spasma,* Thailand) \| MT136160 \| — \| — \| — \| — \| \| *N.* sp. (ex *M. spasma,* Thailand) \| MT136161 \| — \| — \| — \| — \| \| *N.* sp. (ex *M. spasma,* Thailand) \| MT136162 \| — \| — \| — \| — \| \| *N.* sp. (ex *M. spasma,* Thailand) \| MT136163 \| — \| — \| — \| — \| \| *N.* sp. (ex *T. melanopogon,* Thailand) \| MT136165 \| — \| — \| — \| — \| \| *N.* cf. *eradi* (ex *N. arge,* SL) \| — \| — \| KP053776 \| — \| KP053795 \| \| *N.* cf. *eradi* (ex *N. arge,* SL) \| — \| — \| KP053777 \| — \| KP053796 \| \| *N.* sp. (ex *N. grandis,* SL) \| — \| — \| KP053778 \| — \| KP053797 \| \| *N.* sp. (ex *N. grandis,* SL) \| — \| — \| KP053779 \| — \| KP053798 \| \| *N.* sp. (ex *N. macrotis,* SoSu) \| — \| — \| KP053781 \| — \| KP053800 \| \| *N.* sp. (ex *N. macrotis,* SoSu) \| — \| — \| KP053782 \| — \| KP053801 \| \| *N.* sp. (ex *N. macrotis,* SoSu) \| — \| — \| KP053783 \| — \| KP053802 \| \| *N.* sp. (ex *N. macrotis,* SoSu) \| — \| — \| KP053784 \| — \| KP053803 \| \| *N.* sp. (ex *N. macrotis,* SoSu) \| — \| — \| KP053785 \| — \| KP053804 \| \| *N.* sp. (ex *N. macrotis,* SoSu) \| — \| — \| KP053780 \| — \| KP053799 \| \| *N. houini* (ex *N. nana,* SoSu) \| — \| — \| KP053786 \| — \| KP053805 \| \| *N.* sp. (ex *N. thebaica,* SoSu) \| — \| — \| KP053787 \| — \| KP053806 \| \| *N.* cf. *congolensis* (ex *R. landeri,* SL) \| KP053764 \| — \| KP053790 \| KP053771 \| — \| \| *N. gabonensis (*ex *R. alcyone*, DRC) \| KX090647 \| KX090647 \| — \| — \| — \| \| *N. gabonensis* (ex *Rhinol.* sp., So Su) \| KP053766 \| — \| KP053792 \| KP053773 \| KP053810 \| \| *N. gabonensis* (ex *Rhinol.* sp., SoSu) \| KP053767 \| — \| KP053793 \| KP053774 \| KP053811 \| \| *N.* sp. (ex *R. alcyone*, Cote d´Ivoire) \| KF159720 \| — \| — \| KF159751 \| — \| \| *N.* sp. (ex *R. hildebrandti,* Kenya) \| KP053763 \| — \| KP053788 \| KP053770 \| KP053808 \| \| *N.* sp. (ex *R. fumigatus*, Malawi) \| KT750374 \| — \| — \| — \| — \| \| *N.* sp. (ex *R. hildebrandti,* Mozam.) \| KT750341 \| KT750540 \| KT750652 \| — \| KT750553 \| \| *N.* sp. (ex *R. hildebrandti,* Mozam.) \| KT750391 \| KT750538 \| KT750655 \| — \| KT750556 \| \| *N.* sp. (ex *R. hildebrandti,* Mozam.) \| KT750390 \| KT750539 \| KT750654 \| — \| KT750555 \| \| *N.* sp. (ex *R. hildebrandti,* Mozam.) \| KT750392 \| KT750537 \| KT750656 \| — \| KT750557 \| \| *N.* sp. (ex *R. hildebrandti,* Mozam.) \| KT750393 \| KT750536 \| KT750657 \| — \| KT750558 \| \| *N.* sp. (ex *R. hildebrandti,* Mozam.) \| KT750394 \| KT750535 \| KT750658 \| — \| KT750559 \| \| *N.* sp. (ex *Rhinol.* sp., SoSu) \| KP053765 \| — \| KP053791 \| KP053772 \| KP053809 \| \| *N.* sp. (ex *Rhinol.* sp., SoSu) \| KP053768 \| — \| KP053794 \| KP053775 \| KP053812 \| \| *N.* sp. (ex *R. landeri*, Gabon) \| MK098847 \| MK098855 \| MK098840 \| MK098858 \| — \| \| *N.* sp. (ex *R. landeri*, Gabon) \| MK098844 \| MK098853 \| MK098839 \| MK098860 \| — \| \| *N.* sp. (ex *R.* sp.2, Gabon) \| MK098843 \| MK098852 \| — \| — \| — \| \| *N.* sp. (ex *R.* sp.2, Gabon) \| MK098845 \| — \| MK098842 \| MK098859 \| — \| \| *N.* sp. (ex *R.* sp.2, Gabon) \| MK098846 \| MK098854 \| MK098841 \| — \| — \| \| *N.* sp. (ex *D. cyclops,* Uganda) \| KT750346 \| KT750533 \| KT750660 \| — \| KT750562 \| \| *N.* sp. (ex *D. cyclops,* Uganda) \| KT750395 \| KT750532 \| KT750661 \| — \| KT750563 \| \| *N.* sp. (ex *D. cyclops,* Uganda) \| KT750345 \| KT750531 \| KT750662 \| — \| KT750561 \| \| *N.* sp. (ex *D. cyclops*, Cameroon) Nditam05 \| MZ451639 \| MZ460940 \| — \| MZ460929 \| MZ460937 \| \| *N.* sp. (ex *D. cyclops*, Cameroon) Nditam14 \| MZ451640 \| MZ460945 \| — \| — \| — \| \| *N.* sp. (ex *D. cyclops*, Cameroon) Nditam16 \| MZ451641 \| MZ460944 \| — \| — \| MZ460939 \| \| *N.* sp. (ex *D. cyclops*, Cameroon) Nditam17 \| MZ451642 \| MZ460943 \| — \| MZ460930 \| MZ460938 \| \| *N.* sp. (ex *D. cyclops*, Cameroon) Nditam18 \| MZ451643 \| MZ460942 \| — \| MZ460931 \| — \| \| *N.* sp. (ex *D. cyclops*, Cameroon) Nditam19 \| MZ451644 \| MZ460941 \| — \| — \| — \| \| *N.* sp. (ex *R. landeri*, Cameroon) Nditam01 \| MZ451638 \| MZ460946 \| — \| — \| — \| \| *Polychromo-philus* (Chiroptera) \| *Poly.* murinus (ex *M. daubentonii, Switz.*) \| HM055583 \| MK098850 \| — \| JN990723 \| JN990725 \| \| *Poly. melanipherus* (ex *M. schreibersii,* Switz.) \| JN990709 \| — \| JN990721 \| — \| JN990726 \| \| *Poly.* sp. (ex *M. villiersi,* Guinea) \| KF159681 \| KF159796 \| KF159642 \| KF159731 \| — \| \| *Poly.* sp. (ex *P.* aff. *grandidieri*, Guinea) \| KF159714 \| KF159797 \| KF159639 \| KF159742 \| — \| \|  \| \| \| \| \| \| \| \| **Sample number** \| **Bat species** \| ***cytb*** \| ***fgb*** \| ***acox2*** \| ***rogdi*** \|  \| \| **Nditam15** \| ***Doryrhina cyclops*** \| **—** \| **—** \| **MZ460988** \| **MZ460991** \| \| **Nditam09** \| ***Doryrhina cyclops*** \| **—** \| **—** \| **MZ460990** \| **MZ460992** \| \| **Nditam10** \| ***Doryrhina cyclops*** \| **—** \| **—** \| **MZ460989** \| **—** \| \| **Nditam11** \| ***Rhinolophus* cf. *landeri*** \| **MZ460933** \| **—** \| **—** \| **—** \| \| **Ngambe01** \| ***Epomophorus pusillus*** \| **MZ460934** \| **—** \| **—** \| **—** \| \| **Nditam02** \| ***Epomophorus pusillus*** \| **MZ460935** \| **—** \| **—** \| **—** \| \| **Yoko08** \| ***Epomophorus pusillus*** \| **MZ460936** \| **—** \| **—** \| **—** \| \| **Ese04** \| ***Epomophorus pusillus*** \| **—** \| **MZ451636** \| **—** \| **—** \| \| **Ese06** \| ***Epomophorus pusillus*** \| **MZ460932** \| **MZ451637** \| **—** \| **—** \|   Mozam. = Mozambique, Switz. = Switzerland; SL = Sierra Leone, SoSu = South Sudan |
| --- | --- | --- | --- | --- | --- | --- | --- | --- | --- | --- | --- | --- | --- | --- | --- | --- | --- | --- | --- | --- | --- | --- | --- | --- | --- | --- | --- | --- | --- | --- | --- | --- | --- | --- | --- | --- | --- | --- | --- | --- | --- | --- | --- | --- | --- | --- | --- | --- | --- | --- | --- | --- | --- | --- | --- | --- | --- | --- | --- | --- | --- | --- | --- | --- | --- | --- | --- | --- | --- | --- | --- | --- | --- | --- | --- | --- | --- | --- | --- | --- | --- | --- | --- | --- | --- | --- | --- | --- | --- | --- | --- | --- | --- | --- | --- | --- | --- | --- | --- | --- | --- | --- | --- | --- | --- | --- | --- | --- | --- | --- | --- | --- | --- | --- | --- | --- | --- | --- | --- | --- | --- | --- | --- | --- | --- | --- | --- | --- | --- | --- | --- | --- | --- | --- | --- | --- | --- | --- | --- | --- | --- | --- | --- | --- | --- | --- | --- | --- | --- | --- | --- | --- | --- | --- | --- | --- | --- | --- | --- | --- | --- | --- | --- | --- | --- | --- | --- | --- | --- | --- | --- | --- | --- | --- | --- | --- | --- | --- | --- | --- | --- | --- | --- | --- | --- | --- | --- | --- | --- | --- | --- | --- | --- | --- | --- | --- | --- | --- | --- | --- | --- | --- | --- | --- | --- | --- | --- | --- | --- | --- | --- | --- | --- | --- | --- | --- | --- | --- | --- | --- | --- | --- | --- | --- | --- | --- | --- | --- | --- | --- | --- | --- | --- | --- | --- | --- | --- | --- | --- | --- | --- | --- | --- | --- | --- | --- | --- | --- | --- | --- | --- | --- | --- | --- | --- | --- | --- | --- | --- | --- | --- | --- | --- | --- | --- | --- | --- | --- | --- | --- | --- | --- | --- | --- | --- | --- | --- | --- | --- | --- | --- | --- | --- | --- | --- | --- | --- | --- | --- | --- | --- | --- | --- | --- | --- | --- | --- | --- | --- | --- | --- | --- | --- | --- | --- | --- | --- | --- | --- | --- | --- | --- | --- | --- | --- | --- | --- | --- | --- | --- | --- | --- | --- | --- | --- | --- | --- | --- | --- | --- | --- | --- | --- | --- | --- | --- | --- | --- | --- | --- | --- | --- | --- | --- | --- | --- | --- | --- | --- | --- | --- | --- | --- | --- | --- | --- | --- | --- | --- | --- | --- | --- | --- | --- | --- | --- | --- | --- | --- | --- | --- | --- | --- | --- | --- | --- | --- | --- | --- | --- | --- | --- | --- | --- | --- | --- | --- | --- | --- | --- | --- | --- | --- | --- | --- | --- | --- | --- | --- | --- | --- | --- | --- | --- | --- | --- | --- | --- | --- | --- | --- | --- | --- | --- | --- | --- | --- | --- | --- | --- | --- | --- | --- | --- | --- | --- | --- | --- | --- | --- | --- | --- | --- | --- | --- | --- | --- | --- | --- | --- | --- | --- | --- | --- | --- | --- | --- | --- | --- | --- | --- | --- | --- | --- | --- | --- | --- | --- | --- | --- | --- | --- | --- | --- | --- | --- | --- | --- | --- | --- | --- | --- | --- | --- | --- | --- | --- | --- | --- | --- | --- | --- | --- | --- | --- | --- | --- | --- | --- | --- | --- | --- | --- | --- | --- | --- | --- | --- | --- | --- | --- | --- | --- | --- | --- | --- | --- | --- | --- | --- | --- | --- | --- | --- | --- | --- | --- | --- | --- | --- | --- | --- | --- | --- | --- | --- | --- | --- | --- | --- | --- | --- | --- | --- | --- | --- | --- | --- | --- | --- | --- | --- | --- | --- | --- | --- | --- | --- | --- | --- | --- | --- | --- | --- | --- | --- | --- | --- | --- | --- | --- | --- | --- | --- | --- | --- | --- | --- | --- | --- | --- | --- | --- | --- | --- | --- | --- | --- | --- | --- | --- | --- | --- | --- | --- | --- | --- | --- | --- | --- | --- | --- | --- | --- | --- | --- | --- | --- | --- | --- | --- | --- | --- | --- | --- | --- | --- | --- | --- | --- | --- | --- | --- | --- | --- | --- | --- | --- | --- | --- | --- | --- | --- | --- | --- | --- | --- | --- | --- | --- | --- | --- | --- | --- | --- | --- | --- | --- | --- | --- | --- | --- | --- | --- | --- | --- | --- | --- | --- | --- | --- | --- | --- | --- | --- | --- | --- | --- | --- | --- | --- | --- | --- | --- | --- | --- | --- | --- | --- | --- | --- | --- | --- | --- | --- | --- | --- | --- | --- | --- | --- | --- | --- | --- | --- | --- | --- | --- | --- | --- | --- | --- | --- | --- | --- | --- | --- | --- | --- | --- | --- | --- | --- | --- | --- | --- | --- | --- | --- | --- | --- | --- | --- | --- | --- | --- | --- | --- | --- | --- | --- | --- | --- | --- | --- | --- | --- | --- | --- | --- | --- | --- | --- | --- | --- | --- | --- | --- | --- | --- | --- | --- | --- | --- | --- | --- | --- | --- | --- | --- | --- | --- | --- | --- | --- | --- | --- | --- | --- | --- | --- | --- | --- | --- | --- | --- | --- | --- | --- | --- | --- | --- | --- | --- | --- | --- | --- | --- | --- | --- | --- | --- | --- | --- | --- | --- | --- | --- | --- | --- | --- | --- | --- | --- | --- | --- | --- | --- | --- | --- | --- | --- | --- | --- | --- | --- | --- | --- | --- | --- | --- | --- | --- | --- | --- | --- | --- | --- | --- | --- | --- | --- | --- | --- | --- | --- | --- | --- | --- | --- | --- | --- | --- | --- | --- | --- | --- | --- | --- | --- | --- | --- | --- | --- | --- | --- | --- | --- | --- | --- | --- | --- | --- | --- | --- | --- | --- | --- | --- | --- | --- | --- | --- | --- | --- | --- | --- | --- | --- | --- | --- | --- | --- | --- | --- | --- | --- | --- | --- | --- | --- | --- | --- | --- | --- | --- | --- | --- | --- | --- | --- | --- | --- | --- | --- | --- | --- | --- | --- | --- | --- | --- | --- | --- | --- | --- | --- | --- | --- | --- | --- | --- | --- | --- | --- | --- | --- | --- | --- | --- | --- | --- | --- | --- | --- | --- | --- | --- | --- | --- | --- | --- | --- | --- | --- | --- | --- | --- | --- | --- | --- | --- | --- | --- | --- | --- | --- | --- | --- | --- | --- | --- | --- | --- | --- | --- | --- | --- | --- | --- | --- | --- | --- | --- | --- | --- | --- | --- | --- | --- | --- | --- | --- | --- | --- | --- | --- | --- | --- | --- | --- | --- | --- | --- | --- | --- | --- | --- | --- | --- | --- | --- | --- | --- | --- | --- | --- | --- | --- | --- | --- | --- | --- | --- | --- | --- | --- | --- | --- | --- | --- | --- | --- | --- | --- | --- | --- | --- | --- | --- | --- | --- | --- | --- | --- | --- | --- | --- | --- | --- | --- | --- | --- | --- | --- | --- | --- | --- | --- | --- | --- | --- | --- | --- | --- | --- | --- | --- | --- | --- | --- | --- | --- | --- | --- | --- | --- | --- | --- | --- | --- | --- | --- | --- | --- | --- | --- | --- | --- | --- | --- | --- | --- | --- | --- | --- | --- | --- | --- | --- | --- | --- | --- | --- | --- | --- | --- | --- | --- | --- | --- | --- | --- | --- | --- | --- |

**Supplemental Table S3:** Overview of number of investigated bat individuals and infections per sampling sites and season and across sex and age groups

| **Bat species** | **sex** | **age** | **habitat type/sampling location* and season** | | | |
| --- | --- | --- | --- | --- | --- | --- |
|  |  |  | **Forest wet season**  **# infected/# total**  (sampl. location) | **Forest dry season # infected/# total**  (sampl. location) | **Savanna dry**  **# infected/# total**  (sampl. location) | **farm wet season**  **# infected/# total**  (sampl. location) |
| ***Doryrhina cyclops*** | females | ad | **4/6** (L4) | - | - | - |
|  |  | juv | 0/0 (L4) | - | - | - |
|  | males | ad | **2/3** (L4) | - | - | - |
|  |  | juv | 0/1 (L4) | - | - | - |
| *Hipposideros abae* | males | ad | **-** | - | 0/2 (L11) | - |
| *Hipposideros curtus* | females | ad | 0/10 (L5) | - | - | - |
|  |  | juv | 0/6 (L5) | - | - | - |
|  | males | ad | 0/14 (L5) | - | - | - |
|  |  | juv | 0/3 (L5) | - | - | - |
| *Hipposideros fuliginosus* | females | ad | **-** | 0/8 (L3) | - | - |
|  |  | juv | - | 0/1 (L2) | - | - |
|  | males | ad | - | 0/10 (L3) | - | - |
|  |  | juv | - | 0/1 (L3) | - | - |
| *Hipposideros ruber* | females | ad | 0/1 (L5)  0/6 (L6) | - | - | - |
|  |  | juv | 0/4 (L6) | 0/2 (L2) | - | - |
|  | males | ad | 0/1 (L5)  0/6 (L6) | 0/4 (L2) | - | - |
|  |  | juv | 0/1 (L6) | - | - | - |
| *Eidolon helvum* | males | ad | **-** | 0/1(L3) | - | - |
| *Epomops franqueti* | females | ad | 0/10 (L9) | - | - | - |
|  | females | juv | 0/4 (L9) | - | - | 0/1 (L1) |
| ***Epomophorus pusillus*** | females | ad | 0/3 (L9) | **1/1**(L3) | - | **5/5**(L1) |
|  |  | juv | 0/2 (L9) | - | - | **11/11** (L1) |
|  | males | ad | 0/4 (L9) | 0/1 (L3) | - | **2/2** (L1) |
|  |  | juv | - | - | - | **-** |
| *Rousettus aegyptiacus* | female | juv | - | 0/1 (L2) | - | - |
| *Rhinolophus alcyone* | female | ad | - | 0/1 (L3) | - | - |
|  | male | ad | - | 0/1 (L3) | - | - |
| ***Rhinolophus* landeri** | females | ad | 0/2 (L5)  **1/1**(L8) | - | - | - |
|  |  | juv | 0/1 (L5) | - | - | - |
|  | males | ad | 0/2 (L5)  **1/2**(L8) | - | - | - |
|  |  | juv | 0/2 (L8) | - | - | - |
| ***Nycteris grandis*** | male | ad | 1/1 (L7) | - | - | - |
| *Glauconycteris humeralis* | female | ad | 0/1 (L10) | - | - | - |
| *Laephotis nanus* | female | ad | 0/1 (L10) | - | - | - |
|  | male | ad | 0/1 (L10) | - | - | 0/3 (L1) |

*Sampling locations: L1 = N03.87612/E11.42576; L2 =N03.49257/E11.39864; L3 = N05.49204/E11.34643; L4 = N05.21892/E11.14287; L5 = N05.15850/E12.12619; L6 = N0512836/E1133282; L7 = N05.28539/E11.66853; L8 = N05.21891/E11.14285; L9 = N03.64444/E10.77459; L10 = N05.21757/E11.14305; L11 = N05.53087/E11.39769

**Supplemental Table S4:** Overview of infected bat individuals of the study, sampling habitat, sampling season and parasitemia

| **Bat species** | **Sampling Code** | **Habitat types** | **Seasons** | **parasitemia in %** |
| --- | --- | --- | --- | --- |
| *Doryrhina cyclops* | Nditam05 | Forest | Wet season | 0.08 |
| *Doryrhina cyclops* | Nditam14 | Forest | Wet season | 0.05 |
| *Doryrhina cyclops* | Nditam16 | Forest | Wet season | 0.10 |
| *Doryrhina cyclops* | Nditam17 | Forest | Wet season | 0.07 |
| *Doryrhina cyclops* | Nditam18 | Forest | Wet season | 1.02 |
| *Doryrhina cyclops* | Nditam19 | Forest | Wet season | 0.07 |
| *Epomophorus pusillus* | Ngambe01 | Forest | Dry season | 0.03 |
| *Epomophorus pusillus* | Nkol01 | Cultivating farm | Wet season | 0.09 |
| *Epomophorus pusillus* | Nkol02 | Cultivating farm | Wet season | 0.04 |
| *Epomophorus pusillus* | Nkol03 | Cultivating farm | Wet season | 0.07 |
| *Epomophorus pusillus* | Nkol04 | Cultivating farm | Wet season | 0.02 |
| *Epomophorus pusillus* | Nkol05 | Cultivating farm | Wet season | 0.14 |
| *Epomophorus pusillus* | Nkol06 | Cultivating farm | Wet season | 0.07 |
| *Epomophorus pusillus* | Nkol07 | Cultivating farm | Wet season | 0.15 |
| *Epomophorus pusillus* | Nkol10 | Cultivating farm | Wet season | 0.11 |
| *Epomophorus pusillus* | Nkol11 | Cultivating farm | Wet season | 0.37 |
| *Epomophorus pusillus* | Nkol12 | Cultivating farm | Wet season | 0.03 |
| *Epomophorus pusillus* | Nkol14 | Cultivating farm | Wet season | 0.06 |
| *Epomophorus pusillus* | Nkol15 | Cultivating farm | Wet season | 0.08 |
| *Epomophorus pusillus* | Nkol17 | Cultivating farm | Wet season | 0.05 |
| *Epomophorus pusillus* | Nkol18 | Cultivating farm | Wet season | 0.36 |
| *Epomophorus pusillus* | Nkol20 | Cultivating farm | Wet season | 0.10 |
| *Epomophorus pusillus* | Nkol22 | Cultivating farm | Wet season | 0.09 |
| *Epomophorus pusillus* | Nkol23 | Cultivating farm | Wet season | 0.12 |
| *Epomophorus pusillus* | Nkol26 | Cultivating farm | Wet season | 0.04 |
| *Nycteris grandis* | Pack06 | Forest | Wet season | 0.21 |
| *Rhinolophus* cf. *landeri* | Nditam01 | Forest | Wet season | 0.07 |
| *Rhinolophus* cf. *landeri* | Nditam11 | Forest | Wet season | 0.71 |
